# Lower Back Pain Heralding Cauda Equina Syndrome in a Patient With Achondroplasia

**DOI:** 10.7759/cureus.22380

**Published:** 2022-02-19

**Authors:** Ting-Yu Lin, Chun-Sheng Ho, Ke-Vin Chang, Wei-Ting Wu, Levent Ozcakar

**Affiliations:** 1 Physical Medicine and Rehabilitation, Lo-Hsu Medical Foundation, Inc. Lotung Poh-Ai Hospital, Yilan, TWN; 2 Physical Medicine and Rehabilitation, National Taiwan University Hospital Bei-Hu Branch, Taipei, TWN; 3 Physical Medicine and Rehabilitation, Hacettepe University Medical School, Ankara, TUR

**Keywords:** defecation, urinary retention, sacral root, spinal stenosis, dwarf

## Abstract

Achondroplasia is the most common cause of congenital dwarfism and is associated with multiple complications due to impaired skeletal development. Herein, we report a 21-year-old woman with achondroplasia experiencing lower back pain which progressed to lower limb weakness and urinary retention. Magnetic resonance imaging revealed decreased interpedicular distance and severe stenosis of the lumbosacral spinal canal. Cauda equina syndrome was diagnosed and she underwent laminectomy. After decompression, pain, muscle strength and bladder function improved significantly. This case highlights the importance of early recognition of spinal compression in patients with achondroplasia.

## Introduction

Achondroplasia is the leading cause of congenital dwarfism with an incidence between 1/10000 and 1/30000 [1,2]. There is no significant difference in the prevalence between females and males. Fibroblast growth factor receptor 3 (FGFR3) mutation accounts for 95% of the cases - with autosomal dominant inheritance whereby most patients have de novo mutations [1-3]. The phenotypic features encompass short stature (an average height of 130 cm), large head, frontal bossing, depressed nasal bridge, kyphosis and bow legs. Rhizomelia, i.e. short limbs particularly at the proximal segments, is the hallmark of congenital dwarfism [2].

These patients are predisposed to neurological complications due to defective skeletal development. Infants born with achondroplasia may suffer from increased intracranial pressure, hydrocephalus, compression of the medulla and cervical spinal cord as a result of narrowed foramen magnum. Respiratory depression and quadriplegia ensue in extreme cases [4]. During adulthood, patients are more likely to encounter thoraco-lumbar spinal stenosis. We hereby report a young lady with achondroplasia who initially presented with lower back pain where the clinical scenario was complicated with cauda equina syndrome.

## Case presentation

A 21-year-old woman with achondroplasia had received epiphyseal stapling of the left distal femur and proximal tibia in 2009 and 2010 for genu varum. The patient had no other systemic disease except achondroplasia. She took acetaminophen 500 mg bid for lower limb pain. She underwent bilateral paratubal cystectomy for painful mesothelial cysts in 2020. Lower abdominal pain subsided after the surgery. The patient was obese with a body mass index of 31.9 kg/m^2^. She was totally independent for basic and instrumental activities of daily life before 2021. No assistive device was used for ambulation but she had mild lower limb weakness after walking for 15 minutes. Urination and defecation functions were normal.

**Figure 1:**
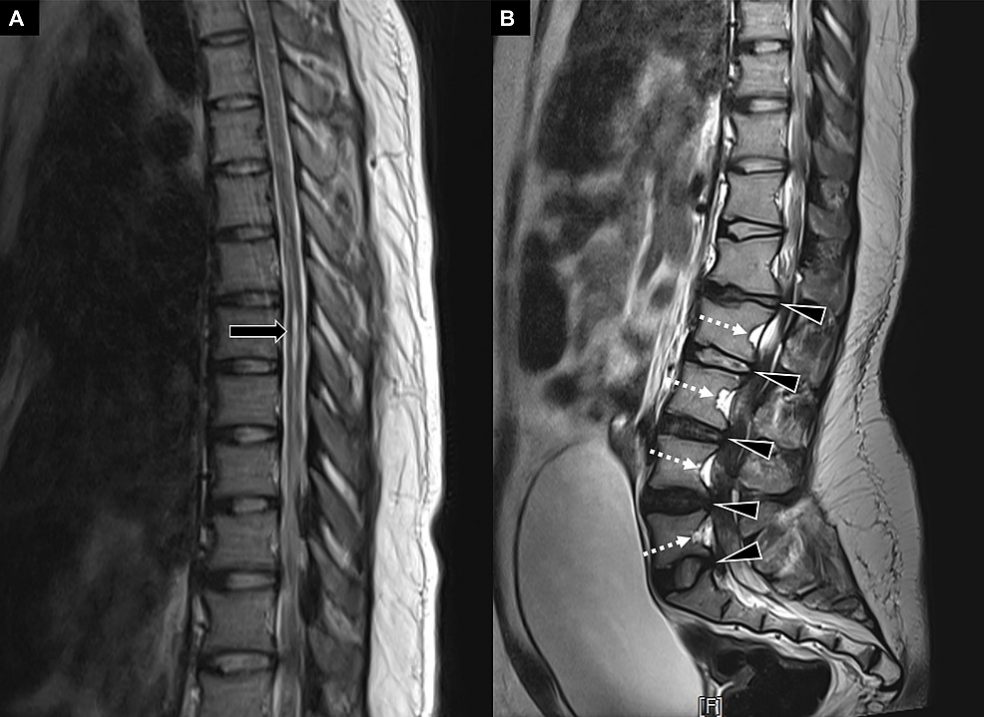
Magnetic resonance imaging for the spine in the sagittal plane Magnetic resonance imaging (T2-weighted sagittal view) shows a syrinx (arrow) at the level of the 6th - 8th thoracic vertebrae (A). Also note the excessive lumbar lordosis, posterior scalloping of vertebral bodies (dashed arrows), protruding intervertebral discs (arrowheads), and lumbar spinal stenosis (B).

Lower back pain developed in early September 2021. Later on, her pain increased and radiated to the left posterior thigh. She also had numbness and prickling pain while stepping on the floor. By the time, the patient could no longer walk on her own due to severe lower extremity weakness. As the difficulty in voiding and defecation also gradually appeared, she visited the emergency department. Vital signs were stable upon triage. Physical examination yielded bilateral reduced muscle strength (2/5) in the lower limbs. Cranial nerve and speech functions were normal. Light reflexes of both eyes were prompt and symmetric. No visual field defect was detected by the confrontation test. There was no limitation of extraocular movements. Facial sensation and movements were normal. Muscle strength of bilateral sternocleidomastoid muscles was full. The patient had midline tongue protrusion and normal phonation. Deep tendon reflexes were normal in the upper limbs but decreased in the lower limbs. Babinski sign was negative. Pinprick and light-touch sensation were markedly impaired below the hip level. Liver and kidney function tests were normal. Magnetic resonance imaging revealed a syrinx across the 6th and 8th thoracic levels (Figure 1A), posterior vertebral scalloping, decreased interpedicular distance and short pedicles of the lumbar spine (Figure 1B) [5,6].

**Figure 2:**
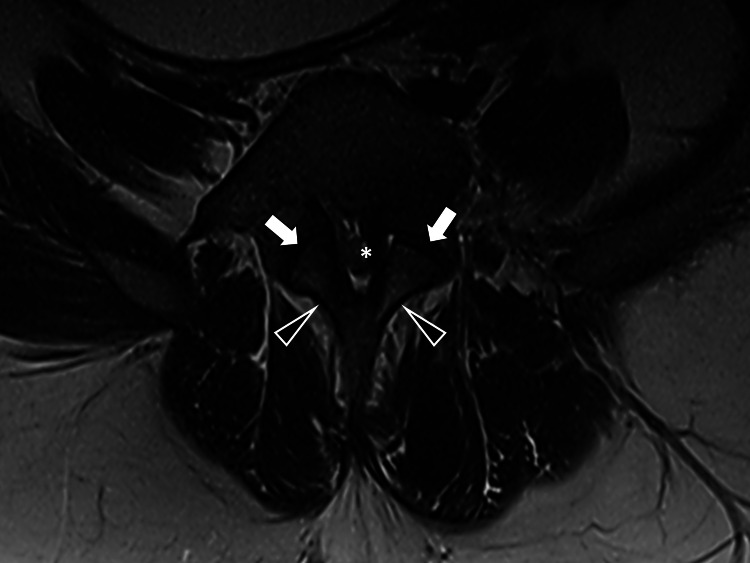
Magnetic resonance imaging for the spine in the axial plane Magnetic resonance imaging (T2-weighted axial view) at the level of L5 shows short thickened pedicles (arrows), extremely decreased transverse diameter of the spinal canal (asterisk) and thickened laminae (arrowheads).

Severe lumbosacral stenosis was apparent on axial images (Figure 2).

Under the impression of cauda equina syndrome, intravenous dexamethasone was administered [7]. Subsequently, she underwent laminectomy (L1-5) and transformational lumbar interbody fusion.

The rehabilitation program started promptly after the surgery. She was under in-patient rehabilitation for three months post-operatively. At first, she received strengthening, bed mobility and transfer training on a mat. In addition, standing table training was given. One month after the surgery, bilateral anterior ankle-foot-orthoses were fabricated for insufficient ankle dorsiflexion and she started standing and ambulation training with a walker. A knee gaiter was used temporarily for left knee buckling and was removed after the second month. Upon discharge, she was independent for transfers and could walk with a walker for 30 meters. Pain management was initiated by using oral mecobalamin, clonazepam, tramadol and acetaminophen and then tapered off within two months post-operatively. In due course, residual urine volume decreased, allowing the removal of the Foley catheter. Muscle strength gradually improved (4/5) in hip flexors, knee extensors, ankle dorsiflexors, long toe extensors and ankle plantar flexors. Eventually, she had a good sitting/standing balance and could ambulate with a walker.

## Discussion

Symptomatic spinal stenosis is seen in approximately 20-30% of patients with achondroplasia and one-third of the affected individuals require surgeries over time. The cumulative prevalence of spinal stenosis increases with age [6,8]. The signs of spinal stenosis often appear before the 3rd decade - in contrast to the general population i.e. >60 years of age [9]. Cervical stenosis is often observed radiographically in achondroplastic children while thoraco-lumbar stenosis becomes more evident in adults.

Patients with achondroplasia have abnormal endochondral ossification which results in short thickened pedicles, thickened lamina and finally narrowing of the spinal canal/foramina [5]. As the interpedicular distance tends to decrease caudally, the space left for the neural content gradually becomes smaller toward the distal spine. Of note, most children and adults with achondroplasia demonstrate disproportionate lumbosacral lordosis due to excessive pelvic tilt and hip flexion contracture. Further, the exaggerated curvature also squeezes the spinal canal [10]. In this aspect, previous studies identified decreased interpedicular distance and kyphosis as the predictors of symptomatic lumbar stenosis [6,8]. Moreover, vertebral malalignment can also cause disc protrusion and spur formation, making patients increasingly susceptible to nerve compression [3,11]. Neurogenic claudication is often the first sign of spinal cord/root impingement - possibly accompanied by sensory impairment. Symptoms may ultimately progress to incontinence or paraplegia [10,12]. Laminectomy relieves the compression and prevents permanent damage which is crucial for long-term walking ability [13].

Importantly, chronic low back pain is present in the majority of achondroplastic patients with symptoms similar to spinal stenosis. The complaint becomes more common as patients grow older [8], affecting 70% of the patients after 50 years of age [14]. Indisputably, pain can take a toll on one’s ability to ambulate and perform daily tasks [15], and therefore warrants optimal management. For sure, the first step should be correct identification of the particular cause. Although back pain is frequently recognized as the presenting symptom of spinal stenosis, also considering the multiple musculoskeletal disorders of this population, not all achondroplasia cases with back pain suffer spinal stenosis. Similar to other low back patients, decreased pain upon spinal flexion and increased lower limb involvement should prompt physicians as regards neurological compromise and the need for appropriate imaging [10,16].

Cauda equina syndrome is a rare but potentially debilitating disorder. While intervertebral disc compression is the most widely documented culprit, several other causes such as spinal stenosis, tumor, cyst, infection, trauma have been described as well. Needless to say, patients with achondroplasia, who frequently have spinal stenosis, are at great risk of cauda equina syndrome. Clinical symptoms are characterized by lower back pain, lower extremity weakness and sensory loss, sciatica, saddle anesthesia, bladder and bowel dysfunction [7]. Since its onset is often vague and there is no set sequence of symptoms, clinicians should stay highly alert concerning the red flags, particularly perineal pain and urinary retention. A comprehensive neurological examination for any sensory/motor loss or reflex changes should be performed. Digital rectal examination and bladder sonography could be considered to evaluate anal tone and urinary retention [17]. Clinicians should proceed with emergent magnetic resonance imaging for final diagnosis [18]. Likewise, medical/surgical decompression should be carried out as early as possible to maintain bowel, bladder and sexual functions also to treat pain [19,20]. It is noteworthy that urinary retention at surgery serves as the strongest predicting factor for post-operative prognosis [19].

## Conclusions

Achondroplasia is well-known for its neurological complications secondary to spinal stenosis whereby cauda equina syndrome is exceptionally dangerous. Accurate diagnosis relies on thorough history taking, detailed physical examination and appropriate imaging. Overall our case highlights the importance of early recognition and prompt management of relevant scenarios in patients with achondroplasia.

## References

[1] Horton WA, Hall JG, Hecht JT (2007). Achondroplasia. Lancet.

[2] Baujat G, Legeai-Mallet L, Finidori G, Cormier-Daire V, Le Merrer M (2008). Achondroplasia. Best Pract Res Clin Rheumatol.

[3] Ornitz DM, Legeai-Mallet L (2017). Achondroplasia: development, pathogenesis, and therapy. Dev Dyn.

[4] Yamada H, Nakamura S, Tajima M, Kageyama N (1981). Neurological manifestations of pediatric achondroplasia. J Neurosurg.

[5] Ferrante L, Acqui M, Mastronardi L, Celli P, Fortuna A (1991). Stenosis of the spinal canal in achondroplasia. Ital J Neurol Sci.

[6] Kahanovitz N, Rimoin DL, Sillence DO (1982). The clinical spectrum of lumbar spine disease in achondroplasia. Spine (Phila Pa 1976).

[7] Gitelman A, Hishmeh S, Morelli BN (2008). Cauda equina syndrome: a comprehensive review. Am J Orthop (Belle Mead NJ).

[8] Fredwall SO, Maanum G, Johansen H, Snekkevik H, Savarirayan R, Lidal IB (2020). Current knowledge of medical complications in adults with achondroplasia: a scoping review. Clin Genet.

[9] Suri P, Rainville J, Kalichman L, Katz JN (2010). Does this older adult with lower extremity pain have the clinical syndrome of lumbar spinal stenosis?. JAMA.

[10] Hoover-Fong J, Cheung MS, Fano V (2021). Lifetime impact of achondroplasia: current evidence and perspectives on the natural history. Bone.

[11] Ireland PJ, Pacey V, Zankl A, Edwards P, Johnston LM, Savarirayan R (2014). Optimal management of complications associated with achondroplasia. Appl Clin Genet.

[12] Schkrohowsky JG, Hoernschemeyer DG, Carson BS, Ain MC (2007). Early presentation of spinal stenosis in achondroplasia. J Pediatr Orthop.

[13] Carlisle ES, Ting BL, Abdullah MA (2011). Laminectomy in patients with achondroplasia: the impact of time to surgery on long-term function. Spine (Phila Pa 1976).

[14] Hunter AG, Bankier A, Rogers JG, Sillence D, Scott CI Jr (1998). Medical complications of achondroplasia: a multicentre patient review. J Med Genet.

[15] Alade Y, Tunkel D, Schulze K (2013). Cross-sectional assessment of pain and physical function in skeletal dysplasia patients. Clin Genet.

[16] Ain MC, Abdullah MA, Ting BL, Skolasky RL, Carlisle ES, Schkrohowsky JG, Rigamonti D (2010). Progression of low back and lower extremity pain in a cohort of patients with achondroplasia. J Neurosurg Spine.

[17] Greenhalgh S, Finucane L, Mercer C, Selfe J (2018). Assessment and management of cauda equina syndrome. Musculoskelet Sci Pract.

[18] Balasubramanian K, Kalsi P, Greenough CG, Kuskoor Seetharam MP (2010). Reliability of clinical assessment in diagnosing cauda equina syndrome. Br J Neurosurg.

[19] Chau AM, Xu LL, Pelzer NR, Gragnaniello C (2014). Timing of surgical intervention in cauda equina syndrome: a systematic critical review. World Neurosurg.

[20] DeLong WB, Polissar N, Neradilek B (2008). Timing of surgery in cauda equina syndrome with urinary retention: meta-analysis of observational studies. J Neurosurg Spine.

